# Evaluation of the association between presenteeism and perceived availability of social support among hospital doctors in Zhejiang, China

**DOI:** 10.1186/s12913-020-05438-5

**Published:** 2020-07-02

**Authors:** Xiaoyu Xi, Qianni Lu, Mengqing Lu, Ailin Xu, Hao Hu, Carolina Oi Lam Ung

**Affiliations:** 1 The Research Center of National Drug Policy& Ecosystem, China Pharmaceutical University, Nanjing, China; 2 State Key Laboratory of Quality Research in Chinese Medicine, Institute of Chinese Medical Sciences, University of Macau, Macau, China

**Keywords:** China, Presenteeism, Doctors, Hospital, Social support

## Abstract

**Background:**

This study investigated the association between presenteeism and the perceived availability of social support among hospital doctors in China.

**Methods:**

A questionnaire was administered by doctors randomly selected from 13 hospital in Hangzhou China using stratified sampling. Logit model was used for data analysis.

**Results:**

The overall response rate was 88.16%. Among hospital doctors, for each unit increase of the perceived availability of social support, the prevalence of presenteeism was decreased by 8.3% (OR = 0.91, *P* = 0.000). In particular, if the doctors perceived availability of appraisal support, belonging support and tangible support as sufficient, the act of presenteeism was reduced by 20.2% (OR = 0.806, *P* = 0.000) 20.4% (OR = 0.803, P = 0.000) and 21.0% (OR = 0.799, P = 0.000) respectively with statistical differences.

**Conclusion:**

In China, appraisal support, belonging support and tangible support, compared to other social support, had a stronger negative correlation with presenteeism among hospital doctors. The benefits of social support in alleviating doctors’ presenteeism warrant further investigation.

## Background

Presenteeism describes the behavior of going to work despite the need to rest after becoming ill [1]. It became a subject of interest since the 1970s and was initially portrayed as a positive organizational behavior as it was a common belief that excellent attendance indicated excellent performance [2]. This was until 1990s when, during the economic depression, a reduced productivity was observed when employees continued to go to work despite being sick. Since then, the conceptualization of presenteeism underwent a drastic turn, and the important implications and impact of presenteeism were considered more carefully. Later, presenteeism has been shown to be a costly problem imposing economic loss at company level impeding the growth of productivity [3, 4]. At individual level, the more frequent presenteeism is practiced, the greater the reduction in their efficiency and the influence on the health has resulted [5–8].

Presenteeism and its implications among doctors are particularly problematic [9–12]. First and foremost, presenteeism was a risk factor for doctors’ own health [1, 13] and future sickness absence (also known as absenteeism) [14]. The viscous cycle was that physical [6, 15] and mental [16–18] health problems would also increase the prevalence of presenteeism. Presenteeism might also affect doctors’ performance at work increasing the risks of medical errors and productivity loss, threatening patients’ well-being especially with their drug treatment [19]. Moreover, it has also been estimated that, across all residents working in the USA, there was an added cost of more than USD 1.2 million attributable to depression related presenteeism, suggesting that residents’ mental illness was a hidden but significant source of healthcare costs nationwide [20].

Social capital has been shown to be an important determination of presenteeism [21, 22]. It is a collective term that refer to the inherent resources contained within the interpersonal relationships which can bring a series of social output [23]. In the modern society, social capital is used very often at individual level to describe the resources one person has within a community [24]. In 2013, a study on 718 employees in two Dutch companies found that organizational social capital was significantly associated with two health indicators (namely perceived health and emotional exhaustion) which were in turn associated with presenteeism [21]. In 2018, a multilevel study in China further demonstrated that workplace social capital at individual-level and workplace-level were both negatively associated with presenteeism [25].

Being a component of social capital, the perception of social support has been used to measure the perceived availability of social support by an individual [26–30]. In theory, the perceived social support may affect the prevalence of presenteeism through its impact on the individual’s health [31]. In terms of physical health, perceived social support was a significant predictor of lower systolic and diastolic blood pressure [32] and was found to be associated with improved health-related quality of life [33]. In terms of mental health, perceived social support had a close association with a person’s personality [34], posed important consequences on the well-being in theory [35] and in practice [32]. A growing body of evidence suggests that social support may have a role to play in alleviating presenteeism although indirectly [36–38]. A study in 2015 that used Japanese IT employees as study objects suggested that presenteeism could be predicted by a lack of workplace social support [36]. A number of recent studies on health workers in China also supported the findings about the significant indirect effects between supervisor support and presenteeism, and between coworker support and presenteeism [37, 38]. In these studies, it was found that coworker support had a significant inverse effect on presenteeism, and collectively with supervisor support could improve organizational commitment.

In China, the prevalence of presenteeism among doctors was found to be consistently higher, ranging from 47.5% for primary medical staff [39] to 66.4% for hospital doctors [40] as compared to as low as 15% among other employees [41]. Hospital doctors are especially prone to presenteeism mainly due to high job demand and intense occupational stress [41]. There is no sign for the situation to improve considering the continuous doctor shortage [42], deterioration of doctor-patient relationship [43], long working hours [44], heavy workloads [45] especially after the implementation of the recent health reform. Collectively, these may make it more challenging for hospital doctors to resist presenteeism. While previous studies have provided a basic understanding about the association between presenteeism and perceived social support among company employees and healthcare workers in general, little has been specifically reported about that among doctors and hospital doctors in China. Therefore, this study aimed to investigate the association between presenteeism and perceived social support among doctors working at healthcare institutes in China. The findings will help improve the current understanding about the association between social support and presenteeism, and more specifically how social support was associated with presenteeism among doctors. It is also anticipated that the findings can help inform actions needed to alleviate presenteeism and raise awareness about the health status for hospital doctors in China.

## Methods

A cross-sectional survey study was conducted in Hangzhou city, Zhejiang Province, China in October 2017. The online questionnaire was hosted by the online questionnaire distribution company “Athena”. Ethical approval was obtained from the Ethics Committee of the China Pharmaceutical University (Project number: CPU2018016).

### Sampling

The Yangtze River Delta is a region in China with a relatively developed economy, high medical standards. By the end of 2016, there were 31,548 healthcare institutions in Zhejiang Province, which is the highest number of medical institutions at province level. Zhejiang Province was, therefore, selected to be the research area of this study. According to the “2016 Zhejiang Health and Family Planning Yearbook” [46], Hangzhou being the provincial capital had 244 hospitals in which there were 34,832 practicing (assisting) physicians. It is the city with the largest number of hospitals and the largest number of physicians in Zhejiang Province. Therefore, Hangzhou was chosen as the sampling city.

Stratified sampling was conducted as followed: firstly, the healthcare institutions were categorized into 3 categories based on the “Hospital Grading Standards” and in consultation with the Hospital Grading Information System in China: basic hospitals, secondary hospitals, and tertiary hospitals (and their overall scales were rated as small, medium and large respectively). Due to their small scale and the small number of healthcare responsibilities, basic hospitals were excluded from this study. Furthermore, for the secondary hospitals and tertiary hospitals, the number of hospitals at each grade was multiplied by the proportion of in the total number of hospitals to yield the hospital sampling number. For instance, there were 15 tertiary hospital and 12 secondary hospitals in Hangzhou, so the hospital sampling number would be 8 and 5 respectively ( $15\times \frac{15}{15+12}=8.3\approx 8$ and $12\times \frac{12}{15+12}=5.3\approx 5$ respectively). Finally, with convenient sampling, a total of 13 hospitals were selected as the primary sampling sites. Within each primary sampling site, sampling was primarily conducted at the key departments, and covered at least half of the departments in each sampling hospital.

In each hospital, doctors were randomly selected by volunteering research assistants who were pharmacy students and had received sufficient research training for the task. Their aim was to randomly selected and collected information from at least 5 doctors from each department.

### Data collection

Before answering the questionnaire, research assistants explained to the participants the research objective and topics, and only provided assistance in answering the questionnaire whenever requested by the participants. All of the research data were collected by team-designed software and were processed into identifiable electronic data. As part of the quality control of the survey, the research team had developed a set of guidelines to help secure good sampling procedure. The survey team was trained prior and during the fieldwork to enhance their adherence to the practice standards prescribed in the guideline. Daily supervision of the survey team and close audit of data entered in the survey software were also performed to identify and resolve any problematic items in the questionnaire and any problems during survey implementation. Systematic check of data quality at different stages of survey study was also conducted to estimate the completeness of the survey study and the frequency of missing data. Collectively, these actions were set to help ensure the quality of data collected in this survey study.

### Questionnaire design

The questionnaire used in this study consisted of 3 parts and was developed in consultation with previous research work. A sample of the questionnaire is provided in the Supplementary Document.

### Part 1 - dependent variable – Presenteeism

Presenteeism is the dependent variable in this study. In order to find out if the participant had practiced presenteeism, the frequency of presenteeism was asked. Referring to the measurement used by scholars such as Johns, Cocker et al., Karanika-Murray et al., a one-question measurement was used to measure the act of presenteeism [1, 47, 48]. Participants were asked “Has it happened over the previous 12 months that you have gone to work despite feeling that you should have taken sick leave because of your state of health?” and had to choose one of the four answers: “never”, “once”, “2–5 times” and “more than 5 times”. Choosing “never” or “once” was considered “no” to presenteeism, and choosing twice or more often was considered “yes” to presenteeism [7, 44]. This measurement has been widely used in previous studies and its reliability has been demonstrated [1, 47, 48].

### Part 2 - independent variable – perceived availability of social support

Interpersonal Support Evaluation List-12 was used to measure 3 aspects of social support including appraisal, belonging and tangible [27]. Interpersonal Support Evaluation List-12 originated from the Interpersonal Support Evaluation List developed by Sheldon Cohen in 1985 [49] and has been used repeatedly adopted in previous studies [50–53]. For each of the 3 included aspects, there were 4 related questions in the questionnaire. Each of the question had four possible answers: “highly disagree”, “possibly disagree”, “possibly agree”, and “highly agree”, with each answer worth 0, 1, 2 and 3 points respectively except for 6 of the questions which had reverse score. The sum of the score of each questions related to each aspect was calculated separately, and totally to give a total score of perceived availability of social support. The higher the score, the better availability of social support perceived by the participants. The scale has been tested and shown to have good reliability [54, 55].

### Part 3 - control variables – demographic information and factors contributing to presenteeism

In addition to the perceived availability of social support that might affect physicians’ act of presenteeism, this study also took into consideration the impact of other possible influencing factors and treated them as control variables. According to Cocker et al., factors which might contribute to presenteeism fell into one of the 4 categories: socio-demographic factors, health factors, financial factors and work-related factors [47]. Socio-demographic factors had 6 items: age, gender, marital status, pregnancy for female participant or for the spouse of male participant, number of children, highest education level). Health factors mainly concerned the history of chronic diseases [15, 56]. Financial factors mainly included the presence of a reward system for full attendance [7, 57] and the participant’s monthly salary [56, 58]. Work-related factors included the number of years the participant had worked at the current hospital [12, 16, 59, 60], their position ranking [61], their job title [16, 59], weekly work hours [7], substitute availability [56, 58], and their superior’s leadership type [62]. All of these information was collected in Part 1. Substitute availability at work was measured using the method developed by Aronsson and Gustafsson [56]. The type of superior’s leadership was measured based on the leadership theory developed by Lewin [63].

The questionnaire design was initially assessed by 5 experienced researchers in public health or occupational health to ensure the theoretical construct was appropriately represented in the questionnaire. To ensure face validity of the questionnaire, these researchers were also asked to evaluate if the questions in the questionnaire would allow reasonable and operational measurements of the dependent variable, independent variables and control variables mentioned above. They were also asked to comment on the face validity of the translation to Chinese. Based on the researchers’ feedback, we revised the translation of two items in the multi-item scales about the perceived availability of social support (Interpersonal Support Evaluation List-12 in Question 2 of the questionnaire) to improve clarity. In a pilot study, the questionnaire was further tested by 8 doctors not included in the sample for readability, clarity and comprehensiveness of the questions. They all came to an agreement that the questions were straight forward and easy to understand. Cronbach’s alpha were also measured to determine the reliability of the multi-item scales related to the perceived availability of social support.

### Statistical analysis

Data collected was organized using Excel initially and data analysis was performed using Stata 14.0. The logit model was used to analyze the association between presenteeism and perceived availability of social support first. In order to more accurately evaluate the impact of perceived availability of social support on presenteeism, the Logit model was used again to further analyze the association of each dimension of perceived availability of social support on presenteeism. Multicollinearity was then checked on each of the 3 dimensions of perceived availability of social support. Appraisal support was set as the dependent variable, and belonging support and tangible support as independent variables. Multiple linear regression was then used to explain the relationship between the dependent variable and the two independent variables. Correlation coefficient was then used to determine the direction and strength of the relationship between the variables. The association between the variables was found to be weak, so the 3 variables were used as separate variables in replacement of the overall perceived availability of social support while the control variables remained unchanged. The Logit model was then used again to test the association between each of the 3 dimensions of perceived availability of social support and presenteeism separately. The correlation was found to be strong, so all 3 dimensions were integrated in the Logit model again which was then run 3 times to analyze the association of each dimension with presenteeism.

## Results

### Participants’ demographic information

A total of 1309 surveys were distributed, 1154 of which were completed giving a response rate of 88.16%. The invited participants who did not participate explained that they did not have the time. The reasons for incompletion were uncertain as the survey was answered anonymously and follow-up questions were, therefore, not feasible. Sociodemographic characteristics included age, gender, marital status, pregnancy status, and their highest education level. Among the participants, 42.37% (*n* = 489) were female. For age stratified, 76.25% (*n* = 880) aged between 30 and 49 years. The sample composition is basically consistent with the overall doctor workforce in China. Further descriptive information of the participants was provided in Table 1.

**Table 1:** Descriptive information of the participants (*n* = 1154)

Variables	Types		Counts	Proportions [%]
**Age**	20–29 years		135	11.70
	30–39 years		439	38.04
	40–49 years		441	38.21
	50–59 years		118	10.23
	60 years or above		21	1.82
**Gender**	Female		489	42.37
	Male		665	57.63
**Marital Status**	Never married		137	11.87
	Married or cohabited		859	74.44
	Divorced or widowed		158	13.69
**Pregnancy status**	Non-pregnant or spouse was not pregnant		1019	88.30
	Pregnancy or spouse was pregnant		135	11.70
**Number of children**	0		385	33.36
	1		484	41.94
	2		237	20.54
	3 or more		48	4.16
**Highest education level**	Junior college		17	1.47
	Bachelor		322	27.90
	Master		603	52.25
	PhD		212	18.37
**History of chronic diseases in the past year**	No		1051	91.07
	Yes		103	8.93
**People management duty**	No		890	77.12
	Yes		264	22.88
**Level of seniority**	None		152	13.17
	Junior staff		313	27.12
	Middle management		275	23.83
	Sub-top management		271	23.48
	Top management		143	12.39
**Weekly work hours**	Less than 34 h		164	14.21
	35–39 h		125	10.83
	40 h		475	41.16
	41–45 h		340	29.46
	46 h or more		50	4.33
**Reward system for full attendance**	No		388	33.62
	Yes		766	66.38
**Substitute availability**	Almost none		221	19.15
	Less than half		521	45.15
	More than half		334	28.94
	All		78	6.76
**Superior’s leadership type**	Authoritarian		249	21.58
	Democratic		638	55.29
	Laissez-faire		267	23.14
	**Minimum**	**Maximum**	**Mean ± SD**	
**Number of years working at the current hospital**	1	50	12.14 ± 8.27	
**Monthly salary [RMB]**	2500	16,000	7920.91 ± 2611.53	

Prevalence of presenteeism and perceived availability of social support.

As shown in Table 2, when asked if they went to work despite feeling that they should have taken sick leave due to their state of health, 66.46% (*n* = 767) of the respondents participants had that experience twice or more over the previous 12 months. The mean score of the overall perceived availability of social support was 17.92 ± 8.14, with appraisal support, belonging support and tangible support scored 6.10 ± 2.78, 5.97 ± 2.93 and 5.85 ± 2.79 respectively. The Cronbach’s α value of the multi-item scales related to Interpersonal Support Evaluation List-12 was 0.804, indicating a satisfactory level of reliability.

**Table 2:** Prevalence of presenteeism and perceived availability of social support (*n* = 1154)

	Types		Counts	Proportions
**Prevalence of presenteeism**	Never or once		387	33.54%
	Twice or more		767	66.46%
	**Minimum**	**Maximum**	**Mean ± SD**	
**Perceived availability of social support**	0	36	17.92 ± 8.14	
appraisal support	0	12	6.10 ± 2.78	
belonging support	0	12	5.97 ± 2.93	
tangible support	0	12	5.85 ± 2.79	

### Logistic regression

There is a strong association between perceived availability of social support and presenteeism (OR = 0.917, P = 0.000), suggesting that the doctors’ perceived social support could be a determinant of their act of presenteeism. Moreover, for each unit increase of the perceived availability of social support, the prevalence of presenteeism would decreased by 8.3% (see Table 3). The R [2] value was 0.3024 indicating an acceptable level of reliability, and the results of a robust test also verified the validity of our model.

**Table 3:** Logistic regression analysis of control factors associated with presenteeism

	Odds Ratio	Robust Std. Err.	z	P	[95% Conf. Interval]	
Perceived availability of social support	0.917	0.012	−6.69	**0.000**	0.893	0.940
Age						
30–39 years	1.188	0.394	0.52	0.604	0.620	2.275
40–49 years	3.718	1.860	2.63	**0.009**	1.395	9.909
50–59 years	6.053	4.734	2.3	**0.021**	1.307	28.037
60 years or above	0.780	0.982	−0.2	0.844	0.066	9.185
Gender	1.394	0.228	2.04	**0.042**	1.012	1.920
Marital status						
Married or cohabited	1.798	0.589	1.79	0.074	0.945	3.418
Divorced or widowed	0.809	0.342	−0.5	0.616	0.353	1.853
Pregnancy status	1.165	0.314	0.56	0.572	0.686	1.976
Number of children						
1	1.120	0.272	0.47	0.640	0.696	1.801
2	2.319	0.732	2.66	**0.008**	1.249	4.307
3 or more	8.653	8.394	2.22	**0.026**	1.292	57.927
Highest education level						
Bachelor	1.443	1.200	0.44	0.659	0.283	7.366
Master	2.241	1.853	0.98	0.329	0.443	11.328
PhD	5.563	4.767	2	**0.045**	1.037	29.832
Chronic disease	0.968	0.289	−0.11	0.912	0.539	1.737
Time working at the current hospital	1.090	0.034	2.79	**0.005**	1.026	1.158
Management duty	0.226	0.056	−6.01	**0.000**	0.139	0.367
Level of seniority						
Junior staff	0.820	0.218	−0.74	0.457	0.487	1.383
Middle management	0.423	0.137	−2.66	**0.008**	0.224	0.798
Sub-top management	1.363	0.630	0.67	0.502	0.551	3.370
Top management	0.580	0.325	−0.97	0.331	0.193	1.738
Weekly work hours						
35–39 h	0.541	0.229	−1.45	0.147	0.236	1.240
40 h	0.750	0.275	−0.78	0.433	0.365	1.541
41–45 h	0.645	0.244	−1.16	0.247	0.307	1.356
46 h or more	0.586	0.301	−1.04	0.298	0.214	1.604
Reward system for full attendance	0.712	0.136	−1.77	0.076	0.490	1.036
Monthly salary [log]	0.295	0.308	−1.17	0.243	0.038	2.293
Lack of substitute availability						
Less than half	0.719	0.179	−1.32	0.185	0.442	1.171
More than half	0.386	0.102	−3.6	**0.000**	0.230	0.648
All	1.180	0.475	0.41	0.681	0.536	2.598
Superior’s leadership type						
Democratic	1.225	0.259	0.96	0.336	0.810	1.853
Laissez-faire	0.800	0.187	−0.95	0.340	0.507	1.265

This study also tried to estimate the association of each of the 3 dimensions of perceived availability of social support and presenteeism: appraisal support, belonging support, and tangible support. Correlation tests showed that the associations between these 3 dimensions and presenteeism were all greater than 0.8, indicating that the associations were both strong and significant. After integrating the 3 dimensions into the logit model for further analysis while the control variables remained unchanged, it was found that all 3 dimensions had a strong negative correlation with presenteeism (*P* = 0.000), and that doctors would reduce their act of presenteeism by 20.2% (OR = 0.806, *P* = 0.000) 20.4%(OR = 0.803, *P* = 0.000) 21.0%(OR = 0.799, *P* = 0.000) if their perceived availability of appraisal support, belonging support and tangible support were sufficiently high respectively (see Table 4).

**Table 4:** Logit regression: presenteesim with appraisal/belonging/tangible support

Correlation test (*** indicates *P* < 0.01)						
	Appraisal support		Belonging support		Tangible support	
**Appraisal support**	1.0000					
**Belonging support**	0.8909***		1.0000			
**Tangible support**	0.8621***		0.8731***		1.0000	
**Logit regression analysis of different types of social support associated with presenteeism**						
	**Odds Ratio**	**Robust Std. Err.**	**Z**	**P > z**	**[95% Conf. Interval]**	
**Appraisal support**	0.806	0.029	−5.97	0.000	0.751	0.865
**Belonging support**	0.803	0.028	−6.30	0.000	0.750	0.860
**Tangible support**	0.799	0.030	−6.02	0.000	0.743	0.860

The study took into consideration of 15 control variables, 8 of which were found significantly associated with presenteeism: age, gender, number of children, highest education level, number of years working at the current hospital, management duty, level of seniority and lack of substitute availability (see Table 3). In terms of age, doctors aged between 40 and 49 years old (OR = 3.718, *P* = 009) and 50–59 years old (OR = 6.053, *P* = 0.021) are more likely to practice presenteeism when compared with doctors aged 20–29 years old; when compared with the doctors aged between 20 and 29 years old, the prevalence of presenteeism among those aged between 30 and 39 years old and those aged 60 years old or over was higher and lower respectively but with no statistical significance. Male doctors were more likely to practice presenteeism when compared to female doctors (OR = 1.394, *P* = 0.042). The number of children a doctor had, the more likely he/she would practice presenteesim, especially in cases of 2 children (OR = 2.319, *P* = 0.008), or 3 children or more (OR = 8.653, *P* = 0.026) when compared to doctors who had no children. The education level of the doctors also played a significant role in affecting their decision about presenteeism, and doctors with PhD degree were more likely to practice presenteeism when compared with those who did not have formal university degree (OR = 5.563, *P* = 0.045). With regards to work-related factors, the longer the doctor worked in the hospital, the more likely he/she would practice presenteeism (OR = 1.090, *P* = 0.005); the doctors with management duties were more likely to practice presenteeism (OR = 0.226, *P* = 0.000); doctors at middle management level were more likely to practice presenteeism when compared with doctors who were not involved in management hierarchy (OR = 0.423, *P* = 0.008); lack of substitute availability resulting in at least half of the workload to catch up after taking sick leave was another significant contributing factor of presenteeism when compared to doctors who could easily find replacement in case of being absent (OR = 0.386, *P* = 0.000). In addition, in order to verify the stability of the testing model, the two variables (monthly salary and superior’s leadership type) were removed and data analysis was re-conducted. Results of logistic regression analysis still showed an association between perceived availability of social support and presenteeism which was of statistical significance (OR = 0.914, *P* = 0.000), indicating that the analysis model was reasonably stable.

## Discussion

In this study, 2 out of 3 hospital doctors had practiced presenteeism at least twice in a year. It was also found that for each unit increase of the perceived social support, there was a decreased of 8.3% in the prevalence of presenteeism. More specifically, when the availability of appraisal support, belonging support and tangible support was deemed sufficient, there was a reduction of at least 20% in the act of presenteeism. These findings have helped improved the current understanding about the association between presenteeism and perceived social support among doctors and can be used to inform the development of the theoretical association between perceived availability of social support and presenteeism.

The high prevalence of presenteeism is multifaceted. Shortage of doctors is a major reason for not being able to find a replacement when they fall ill and need to take sick leave [64–66]. Doctors’ mentality towards their duty of care for patients, their responsibilities as a co-worker and caring for their own health could also contribute to presenteeism [67]. They might be reluctant to take sick leave on the grounds that they would fail the patients and even the healthcare institute [1, 57]. Putting their co-workers in a difficult position if they did not turn up at work even when being sick could pose a sense of guilt [68]. Some doctors believed that sick leave would not help with their recovery, and it was more meaningful to remain in the hospital and look after their patients [67]. The tremendous pressure at work was also another unique factor contributing to presenteeism among doctors [68–71]. Since the healthcare reform kicked start in 2009, public hospitals had become an integral part of the transition and were mandated to establish an operation model with high standards and efficiency that meets the remuneration criteria. Effort-reward imbalance in the doctors’ remuneration system complicated with high demands of the intensity and duration of their performance greatly increased the pressure to continuously work despite feeling sick [72].

At present, scholars had come up with few solutions to alleviate the act of presenteeism among doctors. Through this large-scale investigation, however, a significant negative correlation between the perceived availability of social support and presenteeism among doctors had been identified. The more available social support perceived by the doctors, the less likely they were to practice presenteeism. Based on the findings of this study, providing doctors with sufficient social support will stand a good chance of reducing their act of presenteeism and, thus, the adverse consequences as a result. In particular, considering the associations between each of the 3 dimensions of social support and presenteeism were found significant, fostering the appraisal support, belonging support and tangible support for doctors could be prioritized in solving the presenteeism problems.

Firstly, the availability of appraisal support to the doctors should be enhanced, which could include regular professional conferences so that they could easily grasps the latest updates in medical advancement; the collaboration between doctors and pharmacists should also be encouraged in order to create an environment for team work [73]. Internationally, many models guiding the collaboration between doctors and pharmacists had been developed showing that pharmacist’s professional participation in caring for the patients could be a promising clinical development that benefited both patients and doctors [74–76]. On the other hand, reinforcing of the belonging support could start with team building and improving the doctor-patient relationship. Team building should not only limit to strengthening the bonding among the doctors, but, more importantly, should extend to enhance their mutual awareness of replacement options so they would no longer assume “no-one else do my work” [77].

Facing the challenges of the worsening of the doctor-patient relationship [78], doctors’ perception about the availability of social support is exceptionally important. During the course of improving the doctor-patient relationship, Rogers suggested that doctors should be supported to approach their relationship with the patients from 3 perspectives: congruence, unconditioned positive regard, and empathic understanding. With an endeavor to maintain a positive and trustful relationship with patients, doctors would be more likely to receive understanding and support from patients in return [79]. At last but not least, more tangible support should be made available to the doctors which mainly concerned their remuneration system. Previous studies already showed that disparities in remuneration between different types of doctors could be vast and might impede their overall performance [80]. The findings about the doctors’ salaries from this study also mirrored this observation. Therefore, in order to improve the tangible support of doctors and reduce presenteeism, reasonable adjustment to their remuneration system should take place. The actual working hours of the doctors should matched their expected working hours to allow them maintain a sound work-life balance and spare enough attention to their own health [7]. Overall, the government and the healthcare institutions should pay more attention to the practice of presenteeism by doctors and should start with actions that counteract the foreseeable shrinking supply of physicians [65, 66].

In this study, it was found that the perceived social support was inversely associated with presenteeism among hospital doctors in Zhejiang, China. While such finding improves the current understanding about the association between the 2 variable, further studies are needed to inform future intervention studies on measures alleviating presenteeism. Considering the limitations of the findings from this study, and based on the analysis on perspective research on presenteeism [81], to investigate the needs to incorporate the availability of social support for doctors when managing a healthcare facility, future studies are needed to investigate [1] the long-term and short-term functional and dysfunctional effects of presenteeism [2]; the productivity loss attributable to presenteeism; and [3] the impact of interventions that improve social support on the effects of presenteeism and the productivity loss attributable to presenteeism. Moreover, the subjects in this study were comprised exclusively of hospital doctors in Zhejiang. To inform actions at policy level, future studies targeting doctors working at different healthcare settings and in other regions in China are needed to enhance the understanding of the association between social support and presenteeism about the overall doctor workforce in China. Moreover, due to constraints of questionnaire design, the impact of self-esteem support, which is another measurement of social support, on presenteeism was not evaluated warranting further research in the future.

## Conclusion

In this study, a significant negative association between their perceived availability of social support and presenteeism was identified among hospital doctors in China. Appraisal support, belonging support, and tangible support were also inversely associated with presenteeism on their own right. More social support should be provided to the doctors and more awareness about their individual health should be raised at the levels of health institutions and the government.

## Supplementary information

**Additional file 1.**

## Data Availability

The datasets used and analyzed during the current study can be made available from the corresponding author on reasonable request.

## References

[1] Johns G (2010). Presenteeism in the workplace: a review and research agenda. J Organ Behav.

[2] Lin H, Lu L (2013). Presenteeism in workplace: constructing a cross-cultural framework. J Human Resource Management.

[3] Schultz AB, Chen CY, Edington DW (2009). The cost and impact of health conditions on presenteeism to employers. Pharmacoeconomics.

[4] Vänni K, Neupane S, Nygård CH (2017). An effort to assess the relation between productivity loss costs and presenteeism at work. Int J Occup Saf Ergon.

[5] Glanz BI, Dégano IR, Rintell DJ, Chitnis T, Weiner HL, Healy BC (2012). Work productivity in relapsing multiple sclerosis: associations with disability, depression, fatigue, anxiety, cognition, and health-related quality of life. Value Health.

[6] Isetti D, Meyer T (2014). Workplace productivity and voice disorders: a cognitive interviewing study on presenteeism in individuals with spasmodic dysphonia. J Voice.

[7] Yıldız H, Yıldız B, Zehir C, Aykaç M (2015). The antecedents of Presenteeism and sickness absenteeism: a research in Turkish health sector. Procedia Soc Behav Sci.

[8] Zhou Q, Martinez LF, Ferreira AI, Rodrigues P (2016). Supervisor support, role ambiguity and productivity associated with presenteeism: a longitudinal study. J Bus Res.

[9] Huff J, Ablah E (2016). Stress and Presenteeism among Kansas hospital employees: what stress reduction interventions might hospitals benefit from offering to employees?. J Occup Environ Med.

[10] Veale PM, Vayalumkal JV, McLaughlin K (2016). Sickness presenteeism in clinical clerks: negatively reinforced behavior or an issue of patient safety?. Am J Infect Control.

[11] Cullati S, Cheval B, Schmidt RE, Agoritsas T, Chopard P, Courvoisier DS (2017). Self-rated health and sick leave among nurses and physicians: the role of regret and coping strategies in difficult care-related situations. Front Psychol.

[12] Al Nuhait M, Al Harbi K, Al Jarboa A (2017). Sickness presenteeism among health care providers in an academic tertiary care center in Riyadh. J iinfect Public Health.

[13] Bergström G, Bodin L, Hagberg J, Lindh T, Aronsson G, Josephson M (2009). Does sickness presenteeism have an impact on future general health?. Int Arch Occup Environ Health.

[14] Schultz AB, Edington DW (2007). Employee health and presenteeism: a systematic review. J Occup Rehabil.

[15] Pilette PC (2005). Presenteeism in nursing: a clear and present danger to productivity. J Nurs Adm.

[16] Dhaini SR, Zúñiga F, Ausserhofer D, Simon M, Kunz R, De Geest S, Schwendimann R (2017). Are nursing home care workers' health and presenteeism associated with implicit rationing of care? A cross-sectional multi-site study. Geriatr Nurs.

[17] Mikami A, Matsushita M, Adachi H (2013). Sense of coherence, health problems, and presenteeism in Japanese university students. Asian J Psychiatr.

[18] Skagen K, Collins AM (2016). The consequences of sickness presenteeism on health and wellbeing over time: a systematic review. Soc Sci Med.

[19] Wallace JE, Lemaire JB, Ghali WA (2009). Physician wellness: a missing quality indicator. Lancet.

[20] Rosen T, Zivin K, Eisenberg D, Guille C, Sen S (2018). The cost of depression-related Presenteeism in resident physicians. Acad Psychiatry.

[21] van Scheppingen AR, de Vroome EM, ten Have KC, Bos EH, Zwetsloot GI, van Mechelen W (2013). The associations between organizational social capital, perceived health, and employees' performance in two Dutch companies. J Occup Environ Med.

[22] Agampodi TC, Agampodi SB, Glozier N, Siribaddana S (2015). Measurement of social capital in relation to health in low and middle income countries (LMIC): a systematic review. Soc Sci Med.

[23] Coleman JS (1990). Foundations of social theory.

[24] Dufur MJ, Parcel TL, Troutman KP (2013). Does capital at home matter more than capital at school? Social capital effects on academic achievement. Res Social Stratification Mobility.

[25] Zhu Y, Gao J, Wang J, Yu D, Nie X, Dai J, Fu H (2018). Association between workplace social capital and absolute presenteeism: a multilevel study in a Chinese context. J Occup Environ Med.

[26] Cohen S, Hoberman HM (1983). Positive events and social supports as buffers of life change stress1. J Appl Soc Psychol.

[27] Cohen S, Mermelstein R, Kamarck T, Hoberman HM (1985). Measuring the functional components of social support. Social support: Theory, research and applications.

[28] Rose R (2000). How much does social capital add to individual health?. Soc Sci Med.

[29] Kim D, Subramanian SV, Kawachi I (2006). Bonding versus bridging social capital and their associations with self rated health: a multilevel analysis of 40 US communities. J Epidemiol Community Health.

[30] Barman-Adhikari A, Bowen E, Bender K, Brown S, Rice E (2016). A social capital approach to identifying correlates of perceived social support among homeless youth. Child Youth Care Forum.

[31] Newland J, Furnham A (1999). Perceived availability of social support. Pers Individ Differ.

[32] Bowen KS, Birmingham W, Uchino BN, Carlisle M, Smith TW, Light KC (2013). Specific dimensions of perceived support and ambulatory blood pressure: which support functions appear most beneficial and for whom?. Int J Psychophysiol.

[33] Porter A, Fischer MJ, Brooks D (2012). Quality of life and psychosocial factors in African Americans with hypertensive chronic kidney disease. Transl Res.

[34] Swickert RJ, Hittner JB, Foster A (2010). Big five traits interact to predict perceived social support. Personal Individ Differ.

[35] Sirois FM, Millings A, Hirsch JK (2016). Insecure attachment orientation and well-being in emerging adults: the roles of perceived social support and fatigue. Personal Individ Differ.

[36] Kono Y, Uji M, Matsushima E (2015). Presenteeism among Japanese IT employees: personality, temperament and character, job strain and workplace support, and mental disturbance. Psychology.

[37] Yang T, Lei R, Jin X, Li Y, Sun Y, Deng J (2019). Supervisor support, coworker support and Presenteeism among healthcare Workers in China: the mediating role of distributive justice. Int J Environ Res Public Health.

[38] Yang T, Ma T, Liu P, Liu Y, Chen Q, Guo Y, Zhang S, Deng J (2019). Perceived social support and presenteeism among healthcare workers in China: the mediating role of organizational commitment. Environ Health Prev Med.

[39] Tang N, Han L, Yang P, Zhao Y, Zhang H (2019). Are mindfulness and self-efficacy related to presenteeism among primary medical staff: a cross-sectional study. Int J Nurs Sci.

[40] Xi X, Lu Q, Wo T, Pei P, Lin G, Hu H, Ung COL (2019). Doctor’s presenteeism and its relationship with anxiety and depression: a cross-sectional survey study in China. BMJ Open.

[41] Yu J, Wang S, Yu X (2015). Health risk factors associated with presenteeism in a Chinese enterprise. Occup Med.

[42] Anand, S., Fan, V. Y., Zhang, J., Zhang, L., Ke, Y., Dong, Z., & Chen, L. C. 2008. China's human resources for health: quantity, quality, and distribution. Lancet, 372(9651), 1774–1781. doi: 10.1016/S0140-6736(08)61363-X. 10.1016/S0140-6736(08)61363-X 18930528

[43] Zhou M, Zhao L, Campy KS, Wang S (2017). Changing of China′ s health policy and doctor–patient relationship: 1949–2016. Health Policy Technol.

[44] Wu H, Liu L, Wang Y, Gao F, Zhao X, Wang L (2013). Factors associated with burnout among Chinese hospital doctors: a cross-sectional study. BMC Public Health.

[45] Wen J, Cheng Y, Hu X, Yuan P, Hao T, Shi Y (2016). Workload, burnout, and medical mistakes among physicians in China: a cross-sectional study. Bioscience Trends.

[46] Health Commission of Zhejiang Province 2016. Zhejiang Health Yearbook 2016.http://www.zjwjw.gov.cn/col/col1202372/index.html (accessed 14 July 2019).

[47] Cocker F, Martin A, Scott J, Venn A, Otahal P, Sanderson K (2011). Factors associated with presenteeism among employed Australian adults reporting lifetime major depression with 12-month symptoms. J Affect Disord.

[48] Karanika-Murray M, Pontes HM, Griffiths MD, Biron C (2015). Sickness presenteeism determines job satisfaction via affective-motivational states. Soc Sci Med.

[49] Cohen S, Kamarck T, Mermelstein R (1983). A global measure of perceived stress. J Health Soc Behav.

[50] Perrin PB, Panyavin I, Morlett Paredes A, Aguayo A, Macias MA, Rabago B, Picot SJ, Arango-Lasprilla JC (2015). A disproportionate burden of care: gender differences in mental health, health-related quality of life, and social support in Mexican multiple sclerosis caregivers. Behav Neurol.

[51] Rogers, H. L., Brotherton, H. T., Plaza, S. L. O., Durán, M. A. S., & Altamar, M. L. P. 2015. Depressive and anxiety symptoms and social support are independently associated with disease-specific quality of life in Colombian patients with rheumatoid arthritis. Revista Brasileira de Reumatologia (English Edition), 55(5):406–413. dloi: 10.1016/j.rbre.2015.01.005. 10.1016/j.rbr.2015.01.005 25816759

[52] Sripada RK, Pfeiffer PN, Rauch SA, Bohnert K (2015). Social support and mental health service use among individuals with PTSD in a nationally-representative survey. Psychiatric services (Washington, DC).

[53] Rushton PW, Labbé D, Demers L, Miller WC, Mortenson WB, Kirby RL (2017). Understanding the burden experienced by caregivers of older adults who use a powered wheelchair: a cross-sectional study. Gerontol Geriatric Med.

[54] Merz EL, Roesch SC, Malcarne VL, Penedo FJ, Llabre MM, Weitzman OB, Nacas-Nacher EL, Perreira KM, Gonzalez F, Ponguta LA, Johnson TP, Gallo LC (2014). Validation of interpersonal support evaluation list-12 (ISEL-12) scores among English-and Spanish-speaking Hispanics/Latinos from the HCHS/SOL sociocultural ancillary study. Psychol Assess.

[55] Payne TJ, Andrew M, Butler KR, Wyatt SB, Dubbert PM, Mosley TH (2012). Psychometric evaluation of the interpersonal support evaluation list–short form in the ARIC study cohort. SAGE Open.

[56] Aronsson G, Gustafsson K (2005). Sickness presenteeism: prevalence, attendance-pressure factors, and an outline of a model for research. J Occup Environ Med.

[57] Klein J (2013). Presenteeism, absenteeism and psychosocial stress at work among German clinicians in surgery. Gesundheitswesen (Bundesverband der Arzte des Offentlichen Gesundheitsdienstes (Germany)).

[58] Aronsson G, Gustafsson K, Dallner M (2000). Sick but yet at work. An empirical study of sickness presenteeism. J Epidemiol Community Health.

[59] Graf E, Cignacco E, Zimmermann K, Zúñiga F (2015). Affective organizational commitment in Swiss nursing homes: a cross-sectional study. The Gerontologist.

[60] Godøy A (2016). Profiting from presenteeism? Effects of an enforced activation policy on firm profits. Labour Econ.

[61] Woo JM, Kim W, Hwang TY (2011). Impact of depression on work productivity and its improvement after outpatient treatment with antidepressants. Value Health.

[62] Schmid JA, Jarczok MN, Sonntag D, Herr RM, Fischer JE, Schmidt B (2017). Associations between supportive leadership behavior and the costs of absenteeism and presenteeism: an epidemiological and economic approach. J Occup Environ Med.

[63] Schein EH (1996). Kurt Lewin's change theory in the field and in the classroom: notes toward a model of managed learning. Systems Pract.

[64] Daar DA, Alvarez-Estrada M, Alpert AE (2018). The Latino physician shortage: how the affordable care act increases the value of Latino Spanish-speaking physicians and what efforts can increase their supply. J Racial Ethn Health Disparities.

[65] Li HM, Wei ZZ (2016). Solutions to the physician shortage in padiatrics department. Zhejiang People’s Congness Magazine.

[66] Deng XY (2014). Thoughts of Physician's shortage in China. China Health Human Resources.

[67] Giæver F, Lohmann-Lafrenz S, Løvseth LT (2016). Why hospital physicians attend work while ill? The spiralling effect of positive and negative factors. BMC Health Serv Res.

[68] Schreuder JAH, Roelen CAM, Van der Klink JJL, Groothoff JW (2013). Characteristics of zero-absenteeism in hospital care. Occup Med.

[69] Hansen CD, Andersen JH (2008). Going ill to work–what personal circumstances, attitudes and work-related factors are associated with sickness presenteeism?. Soc Sci Med.

[70] Miraglia M, Johns G (2016). Going to work ill: a meta-analysis of the correlates of presenteeism and a dual-path model. J Occup Health Psychol.

[71] Szymczak JE, Smathers S, Hoegg C, Klieger S, Coffin SE, Sammons JS (2015). Reasons why physicians and advanced practice clinicians work while sick: a mixed-methods analysis. JAMA Pediatr.

[72] Wu D, Wang Y, Lam KF, Hesketh T (2014). Health system reforms, violence against doctors and job satisfaction in the medical profession: a cross-sectional survey in Zhejiang Province, eastern China. BMJ Open.

[73] Pharmaceutique, F. I. 2009. FIP Reference Paper Collaborative Practice. https://fip.org/uploads/database_file.php?id=319&table_id= (accessed 14 July 2019).

[74] Rathbone AP, Mansoor SM, Krass I, Hamrosi K, Aslani P (2016). Qualitative study to conceptualise a model of interprofessional collaboration between pharmacists and general practitioners to support patients' adherence to medication. BMJ Open.

[75] Hale AR, Coombes ID, Stokes J, McDougall D, Whitfield K, Maycock E, Nissen L (2013). Perioperative medication management: expanding the role of the preadmission clinic pharmacist in a single Centre, randomised controlled trial of collaborative prescribing. BMJ Open.

[76] Makowsky MJ, Schindel TJ, Rosenthal M, Campbell K, Tsuyuki RT, Madill HM (2009). Collaboration between pharmacists, physicians and nurse practitioners: a qualitative investigation of working relationships in the inpatient medical setting. J Interprofessional Care.

[77] McKevitt C, Morgan M, Dundas R, Holland WW (1997). Sickness absence and ‘working through’illness: a comparison of two professional groups. J Public Health.

[78] Cui LJ, Ji BL (2016). Ocupational stressor analysis of emergency department physicians. Hosp Admin J Chin PLA.

[79] Rogers CR (1957). The necessary and sufficient conditions of therapeutic personality change. J Consult Psychol.

[80] Leigh JP, Tancredi D, Jerant A, Kravitz RL (2010). Physician wages across specialties: informing the physician reimbursement debate. Arch Intern Med.

[81] Ruhle SA, Breitsohl H, Aboagye E, Baba V, Biron C, Correia Leal C, Dietz C, Ferreira AI, Gerich J, Johns G, Karanika-Murray M, Lohaus D, Løkke A, Lopes SL, Martinez LF, Miraglia M, Muschalla B, Poethke U, Sarwat (2020). “To work, or not to work, that is the question” – recent trends and avenues for research on presenteeism. Eur J Work Organizational Psychol.

