# Supplementary material for: Evaluation of the association between presenteeism and perceived availability of social support among hospital doctors in Zhejiang, China
Source: BMC Health Serv Res. 2020 Jul 2;20:609. doi: 10.1186/s12913-020-05438-5 (PMC7331165; doi:10.1186/s12913-020-05438-5)
Supplement: Supplementary file 1 — Additional file 1. [file 12913_2020_5438_MOESM1_ESM.docx]

**The English translation of the questionnaire design**

**Evaluation of the association between presenteeism and perceived availability of social support among hospital doctors in China**

Q1 Has it happened over the previous 12 months that you have gone to

work despite feeling that you really should have taken sick leave because of your state of health?’

□never □once □2–5 times □over 5 times

Q2 Your attitude toward descriptions below:

|  | definitely false | probably false | probably true | definitely true |
| --- | --- | --- | --- | --- |
| 1.If I wanted to go on a trip for a day [for example, to the country or mountains], I would have a hard time finding someone to go with me. | □ | □ | □ | □ |
| 2. I feel that there is no one I can share my most private worries and fears with. | □ | □ | □ | □ |
| 3. If I were sick, I could easily find someone to help me with my daily chores. | □ | □ | □ | □ |
| 4. There is someone I can turn to for advice about handling problems with my family. | □ | □ | □ | □ |
| 5. If I decide one afternoon that I would like to go to a movie that evening, I could easily find someone to go with me. | □ | □ | □ | □ |
| 6. When I need suggestions on how to deal with a personal problem, I know someone I can turn to. | □ | □ | □ | □ |
| 7. I don't often get invited to do things with others. | □ | □ | □ | □ |
| 8. If I had to go out of town for a few weeks, it would be difficult to find someone who would look after my house or apartment [the plants, pets, garden, etc.]. | □ | □ | □ | □ |
| 9. If I wanted to have lunch with someone, I could easily find someone to join me. | □ | □ | □ | □ |
| 10. If I was stranded 10 miles from home, there is someone I could call who could come and get me. | □ | □ | □ | □ |
| 11. If a family crisis arose, it would be difficult to find someone who could give me good advice about how to handle it. | □ | □ | □ | □ |
| 12. If I needed some help in moving to a new house or apartment, I would have a hard time finding someone to help me. | □ | □ | □ | □ |

Q3 How old are you?

□20-29 □30-39 □40-49 □50-59 □more than 60

Q4 Which is your gender?

□Male □Female

Q5 Which is your marital status?

□Never married □Married or cohabited □Divorced or widowed

Q6 Are you / Is your spouse is pregnant?

□No □Yes

Q7 How many children[under 18 years old] do you have?

□0 □1 □2 □3 or more

Q8 What is your highest education level?

□Junior □College □Bachelor □Master □PhD

Q9-1 have you had any chronic diseases in the past years?

□No □Yes

Q9-2 What is your chronic disease?_____________

Q10 Is there any reward system for full attendance in your hospital?

□No □yes

Q11 What is your monthly salary?______________

Q12 How many years do you work at the current hospital?______________

Q13 What is your level of seniority?

□Junior staff □Middle management □Sub-top management □Top management □None

Q14 Do you have a management duty?

□Yes, I am a leader [presence of staff from the lower levels]

□No, I am not a leader [absence of staff from the lower levels]

Q15 How many hours do you work in a week?

□Less than 34 hours □35-39 hours □40 hours □41-45 hours □46 hours or more

Q16 If you are absent from work for up to a week, what proportion of your tasks must you take up again on your return?

□None or only a small proportion □Somewhat less than half □Somewhat more than half □Virtually all

Q17 What is your superior`s leadership type?

□Authoritarian □Democratic □Laissez-faire
